# Mitral Plasticity: The Way to Prevent the Burden of Ischemic Mitral Regurgitation?

**DOI:** 10.3389/fcvm.2021.794574

**Published:** 2022-01-04

**Authors:** Mattia Vinciguerra, Silvia Romiti, Eleonora Wretschko, Mizar D'Abramo, David Rose, Fabio Miraldi, Ernesto Greco

**Affiliations:** ^1^Department of Clinical, Internal Medicine, Anesthesiology and Cardiovascular Sciences, Sapienza University of Rome, Rome, Italy; ^2^Lancashire Cardiac Centre, Blackpool Victoria Hospital, Blackpool, United Kingdom

**Keywords:** mitral valve, ischemic mitral regurgitation, left ventricle remodeling, mitral plasticity, myocardial infarction

## Abstract

The ischemic impairment of the left ventricular contractility, followed by an adverse remodeling leading to the displacement of the papillary muscles (PMs), increased tethering forces and loss of valve competence has been the long-term accepted definition of ischemic mitral regurgitation (IMR). Over the years, different approaches of management have attempted to address valve regurgitation, nevertheless failing to achieve satisfactory outcomes. Recent studies have observed some structural and molecular changes of the mitral valve (MV), challenging the concept of a bystander passive to the subvalvular involvement. Indeed, the solely mechanical stretch of the PMs, as in the dilated left ventricle because of the aortic valve regurgitation, is not enough in causing relevant MV regurgitation. This setting triggers a series of structural changes called “mitral plasticity,” leaflets increase in their size among others, ensuring an adequate systolic area closure. In contrast, the ischemic injury not only triggers the mechanical stretch on the subvalvular apparatus but is also a powerful promotor of profibrotic processes, with an upregulation of the transforming growth factor (TGF)-β signaling pathway, leading to a MV with exuberant leaflet thickness and impaired mobility. In this article, we revise the concept of IMR, particularly focusing on the new evidence that supports dynamic changes in the MV apparatus, discussing the consequent clinical insights of “mitral plasticity” and the potential therapeutic implications.

## Introduction

Ischemic mitral regurgitation (IMR) is a complex syndrome caused by unbalanced closing and tethering forces, that affects 1.6–2.8 million people in the United States and may complicate the 10–20% of patients with the ischemic heart disease (1, 2).

Several surgical approaches have been described and used over the years to address valve incompetence caused by ischemic injury. Nevertheless, mitral valve (MV) surgery is still associated with unsatisfactory outcomes; MV replacement and MV repair, including restrictive annuloplasty, were associated with a not negligible in-hospital mortality and mostly a high recurrence rate of MR in long-term follow-up (1, 3). The target of the medical therapy has been focused on improving LV contractility, following the long-term accepted idea that FMR depended solely on LV impairment, in the absence of a tailored categorization of IMR etiology and subtypes (4).

Current literature focuses to describe in detail IMR phenotypes, differentiating them according to the direction of the tethering forces on MV leaflets and LV dimensions, in order to offer targeted strategies of approach (5).

In the IMR phenotype with asymmetric tethering of valve leaflets, the altered geometry of MV due to the prevalent restricted motion of the posterior mitral leaflets (PML) may be the main driver of MR progression, in the setting of LV not dilated globally. In contrast, symmetric tethering, involving both MV leaflets are often linked to displacement of PMs and LV dilatation, which leads to a progressive impairment of the LV contractility and subsequent poor prognosis (5).

Nevertheless, MV apparatus and leaflets are not a passive bystander, they have the capacity to counteract mechanical stress triggered by LV dilatation, highlighting that the main driver in MR progression may be searched in the biological pathways activated.

In this article, we revise the concept of IMR, initially reporting the pathophysiology of LV adverse remodeling after AMI or chronic ischemia and then focusing on the new evidence that supports dynamic changes in the MV apparatus, finally discussing the consequent clinical insights of “MV plasticity.”

## Ischemic Left Ventricular Adverse Remodeling

Left ventricular adverse remodeling occurring after acute myocardial infarction (AMI) or chronic ischemia is defined by the loss of normal architecture and function of the cardiac chamber secondary to an ischemic injury. Although the clinical course is typically silent for a long period, symptomatic heart failure (HF) is the main adverse outcome, potentially leading to death.

Left ventricular impaired contractility results from changes first in the molecular pathways, cellular and interstitial arrangement, and consequently size, shape, and function (6, 7).

In the early phases after AMI, an inflammatory response involving infiltration of leukocytes and granulocytes and finalized to remove necrotic cell debris is activated. This early lytic and scavenger phase is followed by an anti-inflammatory reparative phase, which ultimately leads to scar formation (8).

The disorder in the delicate balance at the basis of these molecular mechanisms leads to adverse LV remodeling. Contractility may be affected by both the exuberant proinflammatory response leading to an exacerbation of cytokines expression and proteolytic activity, and by the overactivation of cardiac fibroblasts causing an expansion of the fibrotic area (9, 10).

Infarct expansion might also be promoted by changes in LV pressure and shape during the lytic phase because of the higher vulnerability of the cardiac chamber (8).

In particular, the increased wall stress with the release of matrix metalloproteinases from activated inflammatory cells, causes the degradation of collagen matrix, and the consequent loss of physiological cardiomyocytes junctions.

As an effect of tissue rearrangement, an expansion of the infarcted zone occurs in the absence of additional necrosis leading to wall thinning and regional LV cavity dilatation, further worsened by apposition of collagen by the cardiac fibroblasts (7).

The loss of contractile function, how happens in chronic ischemia, triggers compensatory responses involving the non-infarcted myocardium, to maintain the stroke volume, thus increasing the LV loading (11).

Neurohormonal agents belonging to the adrenergic system and renin-angiotensin-aldosterone system (RAAS) among others, are mainly responsible for adaptive mechanisms that occur during and immediately after AMI; an increased plasma concentration promotes inotropic and chronotropic activity, which ensures respectively cardiac output and blood pressure according to the Frank-Starling mechanism; but worsens infarct size expansion due to long-standing volume overload (12).

The temporary beneficial effects brought by compensatory mechanisms, contribute first to an expansion of the infarcted area and in the long term, to postinfarction LV adverse remodeling (11).

The chronic activation of the autonomic system and renin-angiotensin axis exacerbate ventricular dilatation, triggering eccentric hypertrophy, through re-expression of the fetal gene program (13).

Indeed, the chronic neurohormones concentration, produces a hypertrophic stimulus because of the overexpression of sarcoplasmic contractile units, including non-contractile and contractile proteins identifiable only in the fetal period and in the context of global cellular hyperplasia (13).

Hypertrophy and the lack of adequate perfusion, with a consequent imbalance in the oxygen supply, triggers a vicious circle leading to a proapoptotic phase and adverse ventricular remodeling.

Adverse LV remodeling is typically defined by transthoracic echocardiography as a 15–20% increase in both the LV end-diastolic (LVEDV) and end-systolic (LVESV) volumes vs. baseline (14).

The grade of impaired contractility and the infarct size are valid predictors of adverse remodeling and are crucial in the evolution of the global ventricular dilatation. The involvement of more than 10% of the myocardial mass was indicated as the threshold beyond which the regional infarct expansion is followed by eccentric hypertrophy of non-infarcted myocardium, leading to the change of LV from elliptical to a spherical shape, echocardiographically estimated through three-dimensional sphericity index (EDV/(4/3 × π × (D/2)^3^) (13–15). However, the recent evidence suggests that the relationship is not exactly proportional. Data derived from the cardiac magnetic resonance, although have shown an increased incidence of LV remodeling when infarct size affects more than 18.5% of LV mass, have failed to demonstrate absolute criteria. Approximately only 40% of patients in this group underwent progressive LV adverse remodeling. Therefore, could exist an interindividual variability related to predisposing risk factors and an excessive inflammatory response post-AMI, leading to a change in the architecture of the ventricle (16, 17).

Furthermore, it should be considered the crucial role of the mechanical desynchrony decisive for the progression of LV dysfunction. Besides the structural rearrangement of contractile units, LV desynchrony, caused by balance disorder in ion channels, alteration of intercellular communication, and collagen deposition, is a strong indicator for the remodeling severity (18, 19).

## Mitral Valve Dynamic Structural Changes

Disorder balance caused by LV remodeling between closing and tethering forces, which normally ensures optimal mitral leaflet coaptation, has been the long-accepted origin of IMR (20).

The recent literature has challenged our knowledge regarding IMR, making obsolete the concept that the MV apparatus is only a passive bystander.

The active role played by the MV appears clear when we consider the lack of predictivity emerging from the link between LV dilatation, papillary muscles (PMs) displacement, and severity of MR.

The increased sphericity of the LV and tethering forces are widely observable in the chronic aortic valve regurgitation (AVR); surprisingly showing only a low incidence of functional mitral regurgitation (FMR) (21–23).

First through a necroscopic study, Mautner et al. (24) have documented a significant independent association between LV dilatation and mitral leaflet area. A subgroup analysis showed that AV disease associated with enlargement of the LV cavity, was followed by a compensatory change in the MV morphology. In this group of patients larger and heavier dimensions of the mitral leaflet area, the anterior mitral leaflet (AML) length, and the overall mitral circumference were observed, conversely, this was not observed in a control group of subjects and in those with AV stenosis without LV dilatation.

An increase greater than 30% in the mitral leaflet area, varying proportionally with LV volume and ensuring an adequate systolic closure area, has been shown in the subjects with chronic aortic regurgitation (AR) by Beaudoin et al. (25). In the group of FMR, affected by ischemic cardiomyopathy in more than 80% of cases, although the increase in LV volume was comparable with the group with chronic AR, the authors observed relatively smaller dimensions of the MV; without an adequate enlargement of the leaflet as a request by the increased systolic closure area.

The mechanical elasticity working on the MV, which follows LV cavity enlargement and remodeling with PMs displacement in the setting of chronic AR, may promote an adaptive MV active growth with a slow and compensative evolution. Dal Bianco et al. (26) demonstrated, with a comprehensive experimental evaluation of MV morphology, that PMs tethering leads to a cellular change in the valvular tissue, responsible for the active adaptive mechanisms, later called “MV plasticity.”

Endothelial cells of the leaflets, normally quiescent, stimulated by the mechanical stress of remodeling, may increase the expression of signal factors typical of the embryogenic developmental pathways. This active adaptation depends on the molecular process of the endothelial-mesenchymal trans differentiation (EndMT), detected by the expression of mesenchymal markers such as smooth muscle alpha-actin (α-SMA).

In contrast, the MV plasticity results are often not adequate to compensate for the LV remodeling following AMI, although this latter is a powerful trigger of the process (27). These findings are expressions of the delicate balance underlying the reactivation of the embryogenetic biological pathways; this latter, when excessively stimulated in a concentration and time-dependent manner, lead to prevalent fibrotic changes with an unfavorable adaption of MV apparatus.

The inflammatory response following AMI probably plays a crucial role in the altered adaption. The valve tissues infiltration by the cells from the circulating blood, which ultimately differentiate into macrophages, promotes TGF-β upregulation (28).

Therefore, the altered leaflet adaptation with consequent MR post-MI is caused by a vicious cycle triggered by the dysregulation of the post-MI compensatory mechanisms, RAAS among others, which leads to profibrotic changes in the valvular cells with an excessive TGF-β concentration (27).

The molecular changes associated with the altered valve tissue response include an exuberant induction of α-SMA expression involving the endothelium of valvular leaflets on the atrial side. Differently by the solely mechanical stretching/tethering, a deep interstitial penetration involving the valvular interstitial cells (VICs) is present (27).

The infiltration of α-SMA myofibroblasts cells, with increased endothelial neovascularization induces an extracellular matrix (ECM) remodeling with changes in the collagen composition (27).

The adaptive activation of EndMT that stimulates collagen production by the valvular endothelial cells (VECs) and may allow to counteract the mechanical displacement of PMs, is significantly influenced by the environmental context (29).

The inflammatory response seems to be the main driver of the maladaptive process, although in the absence of clear evidence, leading to excessive Angiotensin II (Ang II) concentration (27, 28).

Angiotensin II stimulates the production of growth factors with the conversion of TGF-β to active TGF-β, the principal profibrotic cytokine, in a concentration-dependent manner, increasing the sensitivity of VICs to the fibrogenic actions of TGF-β (30, 31).

The activation of the TGF-β signaling cascade is progressively amplified by paracrine/autocrine stimulation, involving canonical, non-canonical pathways and the expression of endoglin, a transmembrane receptor that modulates the binding of TGF-β to the receptors (31).

Therefore, the initially compensatory mechanism might become counter-productive leading to thickness mitral leaflets and chordae which fail to compensate demanded increased systolic closure area, ultimately impairing coaptation.

The natural history of this molecular rearrangement and structural changes of the MV after an ischemic injury can potentially provide important clinical implications.

## Therapeutic Implications

Evolving therapies are following the improvements in the knowledge around IMR. From the surgical point of view, subvalvular and surgical mitral plasticity techniques, with the aim to complete the inadequate valvular adaption, are receiving the greater endorsement, counteracting mechanically the progressive displacement of PMs, mainly in the setting of minimally invasive approach (32–37).

In particular, surgical mitral plasticity includes techniques such as second-order chordal cut (CC) and AML, which plan to compensate ineffectiveness of the molecular process. The increased surface area of the coaptation demanded by the restricted mobility and the consequent leaflet malcoaptation in the setting of IMR may be adequately covered augmenting anterior leaflet with a pericardial patch. Nevertheless, a limitation in performing the solely AML technique is represented by the reduced leaflets mobility secondary to excessive chordal tethering, with chordae not sufficiently remodeled in length to avoid restricted mobility, which can hamper a more physiological coaptation (33). These observations have been highlighted by Calafiore et al. (33), who approached MV using CC, AML, and restrictive MV annuloplasty, demonstrating excellent outcomes.

Although basing our knowledge on still limited literature, a surgical approach inclusive of subvalular correction is mandatory to restore the leaflet coaptation. Indeed, besides the improved leaflet mobility with a more physiologic curvature, the use of second-order CC has shown to determine significant benefit also to the LV contractility (38).

The mechanical stress imposed with MV leaflets because of the increased tethering forces only partially explain the consequent tissue impairment, as aforementioned, with the inflammatory response and TGF-β upregulation that play a crucial role. Therefore, a potential additional management strategy could aim to modulate the TGF-β biological pathway. The TGF-β production is significantly stimulated by Ang II in several cell types, working as a growth factor also in the cardiac fibroblasts, binding the AT1 receptor (39, 40).

The fibrogenic effects of TGF-β may potentially be modulated by RAAS inhibitors such as Angiotensin-Converting Enzyme inhibitor (ACEi) and Ang II receptor blocker (ARBs), certain drugs.

A significant reduction in the infarct size has been observed in ischemic-reperfusion models when ACEi or ARBs have been provided early after percutaneous intervention (41).

Yu et al. (42) conducted an animal study to analyze the effects of RAAS inhibitors in modulating histopathologic changes leading to the ventricular remodeling after AMI. They distributed the population in the three groups according to the received drugs, differentiating between ACEi, ARBs, and a combination therapy group. Regardless of the specific drugs, the study revealed a significant reduction of activated myofibroblasts, rather than the control group (no treatment). Interestingly, the therapy based solely on the ARB (valsartan) or in combination with fosinopril (ACEi) was importantly shown to completely prevent pathological collagen deposition after AMI and the decrease TGF-β mRNA expression, as demonstrated by other authors (43–46).

The treatment with ACEi may be limited by different factors: the production of Ang II in a non-ACE–dependent pathways and the major beneficial effects made through bradykinin production rather than by suppressing Ang II pathways, among others (47).

ARBs, in particular Losartan, have demonstrated, through blocking the interaction of Ang II with its AT1 receptor, to decrease the release of latent TGF-β and to modulate signaling through Smad pathway (47, 48). Furthermore, the expression of endoglin, a powerful promoter of TGF-β fibrogenic effects, and the inhibition in the ERK 1/2 signaling for non-canonical production represents the main drivers of the beneficial effects (31, 49–51).

These results were encouraging for a potential effect on MV endothelium when TGF-β stimulates EndMT; whereas initially favorable, as contributing to an increase in the leaflet size minimizing MR after ischemic injury, but disadvantageous when upregulated in the long term as mainly responsible for exuberant leaflet thickness and impaired mobility.

Wylie-Sears et al. (30) observed a useful ability of Losartan in manipulating EndMT, avoiding excessive fibrotic growth of leaflets. Losartan, in clonal VEC blocked TGF-β signaling; the inhibition of the non-canonical pathway through phosphorylation of ERK, in mitral VEC, was successful to regulate positively EndMT.

These molecular findings have been later confirmed in an animal test by Bartko et al. (52); Losartan can modulate profibrotic changes of the tethered MV leaflets post-MI leading to the solely positive adaptive growth.

The expression of EndMT has been studied, assessing the effect of Losartan in shams affected by IMR produced through apical MI and PMs retraction. In the Losartan-treated group a significant reduction in the thickness of the leaflets, decreasing cell proliferation, collagen deposition, and neovascularization have been demonstrated.

The maladaptive fibrotic changes may be the catalytic factor in the vicious cycle triggered between MR and LV remodeling. The therapeutical accessibility of the profibrotic processes in positively shaping MV after ischemic injury might be potentially revolutionary, considering the unsatisfactory results achieved by most of the current approaches.

## Conclusion

The burden of IMR is importantly associated with the impairment in the LV contractility often linked to HF, with an important role in worsening patient prognosis. Although evidence is still limited, medical therapy targeted to modulate TGF-β signaling might open new horizons in the management of MR post-MI. In a not-too-distant future, we can hypothesize of targeted medical therapy, that by promoting mitral plasticity could reduce the incidence of the mitral regurgitation secondary to ischemic cardiomyopathy.

## Author Contributions

MV and EG: conceptualization. MV and SR: writing—original draft preparation. DR, EG, EW, MD'A, and FM: review and editing. All authors contributed to the article and approved the submitted version.

## Conflict of Interest

The authors declare that the research was conducted in the absence of any commercial or financial relationships that could be construed as a potential conflict of interest.

## Publisher's Note

All claims expressed in this article are solely those of the authors and do not necessarily represent those of their affiliated organizations, or those of the publisher, the editors and the reviewers. Any product that may be evaluated in this article, or claim that may be made by its manufacturer, is not guaranteed or endorsed by the publisher.
